# On the Evolution of Additive Manufacturing (3D/4D Printing) Technologies: Materials, Applications, and Challenges

**DOI:** 10.3390/polym14214698

**Published:** 2022-11-03

**Authors:** Ayyaz Mahmood, Tehmina Akram, Huafu Chen, Shenggui Chen

**Affiliations:** 1 School of Mechanical Engineering, Dongguan University of Technology, Dongguan 523808, China; 2 School of Life Science and Technology, University of Electronic Science and Technology, Chengdu 610054, China; 3 School of Art and Design, Guangzhou Panyu Polytechnic, Guangzhou 511483, China; 4 Dongguan Institute of Science and Technology Innovation, Dongguan University of Technology, Dongguan 523808, China; 5 CAS Key Laboratory of Soft Matter Chemistry, Hefei National Laboratory for Physical Sciences at Microscale, Collaborative Innovation Center of Chemistry for Energy Materials (iChEM), Department of Chemistry, University of Science and Technology of China, Hefei 230026, China

**Keywords:** 3D printing, additive manufacturing, 4D printing, polymers, aerospace, biomedical, COVID-19

## Abstract

The scientific community is and has constantly been working to innovate and improve the available technologies in our use. In that effort, three-dimensional (3D) printing was developed that can construct 3D objects from a digital file. Three-dimensional printing, also known as additive manufacturing (AM), has seen tremendous growth over the last three decades, and in the last five years, its application has widened significantly. Three-dimensional printing technology has the potential to fill the gaps left by the limitations of the current manufacturing technologies, and it has further become exciting with the addition of a time dimension giving rise to the concept of four-dimensional (4D) printing, which essentially means that the structures created by 4D printing undergo a transformation over time under the influence of internal or external stimuli. The created objects are able to adapt to changing environmental variables such as moisture, temperature, light, pH value, etc. Since their introduction, 3D and 4D printing technologies have extensively been used in the healthcare, aerospace, construction, and fashion industries. Although 3D printing has a highly promising future, there are still a number of challenges that must be solved before the technology can advance. In this paper, we reviewed the recent advances in 3D and 4D printing technologies, the available and potential materials for use, and their current and potential future applications. The current and potential role of 3D printing in the imperative fight against COVID-19 is also discussed. Moreover, the major challenges and developments in overcoming those challenges are addressed. This document provides a cutting-edge review of the materials, applications, and challenges in 3D and 4D printing technologies.

## 1. Introduction 

Three-dimensional printing, also termed additive manufacturing, is a process of creating three-dimensional objects using a 3D printer guided by a computer or digital file. The best description of 3D printing technology was given by Prof. DeSimone during a TED talk in 2015, that is, “3D printing is actually 2D printing over and over again” [1]. The term stereolithography or 3D printing was first introduced by Prof. Charles Hull in 1983 [2]. Rapid prototyping was a more accurate word for 3D printing in the 1980s and the technology was considered to be only useful for producing functional or aesthetically pleasing prototypes. As of 2022, the term additive manufacturing can be used interchangeably with 3D printing as the accuracy, repeatability, and material variety of the technology have improved to the point where some 3D printing processes are considered viable as industrial production technology. Since its introduction and commercialization, 3D printing has been used extensively in the engineering and manufacturing, healthcare, and aerospace industries, particularly for prototyping and creating lightweight complex shapes and structures [3,4,5,6,7,8,9,10,11,12,13] which are difficult to produce using the traditional methods. Thus, 3D printing is recognized as one of the most revolutionary innovations in contemporary manufacturing. It has significantly impacted the way industrial parts/components and equipment are designed and developed. Manufacturers and researchers may now produce intricate shapes and structures that were previously considered to be impossible to create using the standard manufacturing methods. Three-dimensional printing technology has seen constant improvements and has evolved substantially over the last three decades [5,10,13,14,15,16,17,18,19,20,21,22,23].

A 3D printer moves in three dimensions to create a 3D structures. The term 4D printing refers to the addition of a 4^th^ dimension, time, which essentially means that these 3D printed objects transform and change shape over time under the influence of external stimuli such as water [24,25], light [26,27], heat [28,29], pH [30], electricity, magnetic fields, and so on [31,32,33,34]. The resulting object would be referred to as 4D printed objects which had been printed with a 3D printer, but with using 4D printing materials. Thus, a 4D printing material is essentially a material that can transform or change its shape over time upon exposure to external stimuli. Such a transformation in shape can be achieved by using multiple materials in the printer, each of which shows a different response to the external stimuli. For instance, upon exposure to water, adsorption will occur, resulting in the contraction or expansion of different components. Owing to the correct alignment of the components, the contraction or expansion will give rise to the folding or bending of the whole structure. 

**Figure 1:**
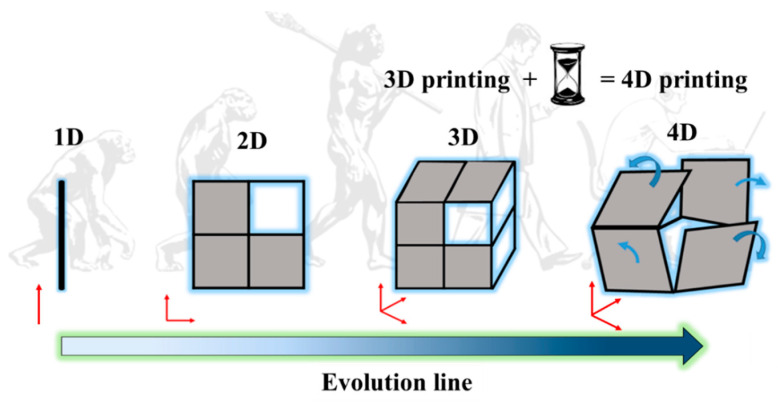
Illustration of the evolution of 1D to 4D printing with time. 4D printing is the addition of time dimension to 3D printing.

The printing process for a 4D object is the same as that of a 3D one. The main difference is that the materials used in 4D printing technology are programmable and undergo a transformation when they come into contact with moisture, light, or heat. Such materials which are responses to external or internal stimuli are referred to as smart materials. Thus, 4D printing essentially uses a 3D printer to print smart materials into the desired objects which then undergo a transformation in structure or property when exposed to external or internal stimuli. The development of multi-material 3D and 4D printing is a current endeavor in the development of AM technologies. It is possible to improve the quality of items using multi-material 3D and 4D printing by changing the composition or kind of materials inside the layers; this is difficult to achieve using traditional production methods. Polymers, metals, ceramics, and biomaterials have all been utilized in various AM processes to create multi-material products. Figure 1 illustrates the evolution line of 1D to 4D printing technology over time.

Despite being a promising technology, 3D and 4D printing have several limitations that prevent them from reaching their full potential. The inability to create complex structures, the unavailability of multi-material printers, the high cost of smart materials and printers, and the lengthy print times are only a few of the industry’s significant obstacles. In this context, in this document, we aim to review the recent advances in 3D and 4D printing technologies, the major differences between the two technologies, the materials used, and their current and potential future applications, including the role of 3D printing in the fight against the recent outbreak of COVID-19. The major difficulties, limitations, and developments in overcoming those challenges are also addressed. Because there are several reviews and publications describing the methods for 3D/4D printing [9,35,36,37,38,39], we will not address the methods but rather focus on the other aspects mentioned above.

**Figure 2:**
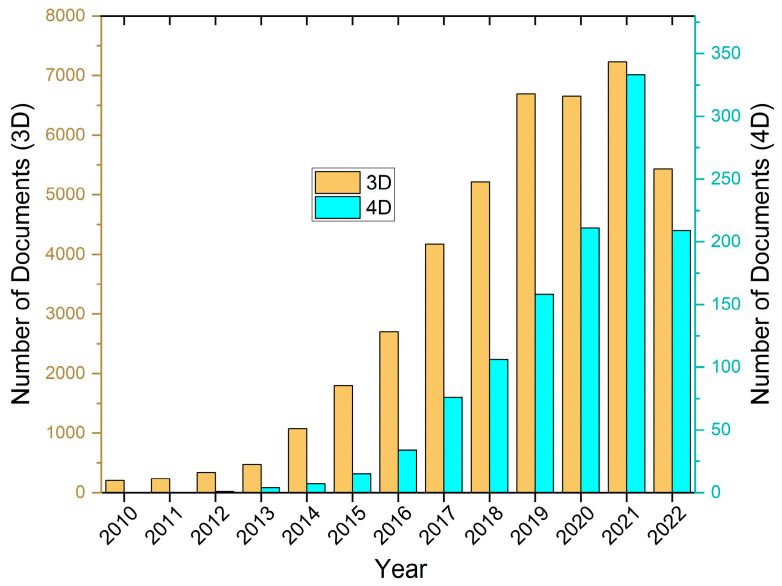
Research published on 3D and 4D printing, in the form of articles, reviews, patents, and conference papers per year as of September 2022. Data extracted from the web of science database. The keywords “additive manufacturing” and/or “3D printing” and “4D printing” were used for exact title search for 3D and 4D printing document search, respectively.

Since 2010, the number of papers studying the additive manufacturing of structures and objects using materials of several types has significantly grown. Figure 2 shows a significant increase in the number of publications on 3D and 4D printing in a diverse selection of journals over the years, which indicates the popularity of this field. According to a feature article that was published in Nature in the year 2020, researchers are working on inventing new methods of printing that are faster and can be used on a larger scale [40]. The journal Science published a number of interesting papers on additive manufacturing in the year 2019, including three investigations on the ultrafast 3D printing of multiscale structures [41,42,43] and two studies on the 3D bioprinting of tissues or organs [44,45]. A variety of materials, including polymers [46], metals [47], ceramics [20,47], glasses [48], biomaterials [49], and multi-material systems [37,50], have been developed for use in AM technologies.

The current paper seeks to give a larger and updated study on the 3D and 4D printing of multi-material components in response to the rising interest in this field by compiling a thorough list of multi-material additive manufacturing techniques that have been reported in the literature.

## 2. Three-Dimensional/Four-Dimensional Printing Process and Materials

### 2.1. General Process

The main printing process is often the same regardless of the method that a 3D/4D printer employs to create objects. The main steps involved in any AM printing technology are briefly described below [15]. The steps are as follows:

- Computer-aided Design (CAD): CAD software creates a three-dimensional model of the object. Using scientific data on the particular materials, the program may produce virtual simulations of how the thing would perform under various circumstances. The CAD model is next converted into Standard Tessellation Language (STL) conversion. STL is a file format that was designed for 3D systems in 1987 by their stereolithography apparatus (SLA) machines. STL files are supported by almost all 3D printers.
- STL file manipulation and the transfer to machines: the STL file is then copied by the user to the computer that manages the 3D printer. There, the user may choose the printing’s size and direction. This is comparable to setting up a usual paper printer to print on both sides or in landscape rather than the portrait orientation.
- The next step is to set up the machine. Each machine has specific guidelines for getting ready for a fresh print job. This entails restocking the printer’s polymers, binders, and other consumables.
- Building process: at this point, you may sit back and relax, as the majority of the building process is handled automatically. Although it may be considerably thinner or thicker, each layer is typically 0.1 mm thick. This procedure might take hours or even days to complete, depending on the size of the item, the machine, and the materials utilized.
- Post-processing: the printed object from many 3D printers will need some post-processing finishing. This can include washing the printed item to get rid of any residual powder or brushing off any water-soluble supports. Since certain materials require time to cure at this stage, the printed structure could be fragile, thus care may be needed to prevent it from breaking or disintegrating. Applications are the next steps, where printed structures/objects are installed and put to use.

The need to print intricate structures at a high resolution has fueled the development of additive manufacturing (AM) techniques. One of the main reasons for the development of AM technologies is the capacity to print massive structures, reduce printing flaws, and improve the mechanical qualities. Fused deposition modeling (FDM) is the most popular 3D printing technique that primarily makes use of polymer filaments. The primary techniques for additive manufacturing (AM) include inkjet printing (IP), contour crafting, stereolithography (SLA), direct energy deposition (DED), laminated object manufacturing (LOM), selective laser sintering (SLS), selective laser melting (SLM), and liquid binding in 3D printing. These techniques are briefly defined, their uses and appropriate materials, and their advantages and disadvantages are presented in Table 1. In Bhushan and Caspers book [51], these techniques are covered in great detail. Mao et al. [52] presented the novel developing techniques for particular applications, such as two-photon polymerization (TPP), projection micro stereolithography (PSLA), and electrohydrodynamic printing (EHDP), while Changhai et al. [53] described the non-contact micro- and nano-printing techniques.

**Figure 3:**
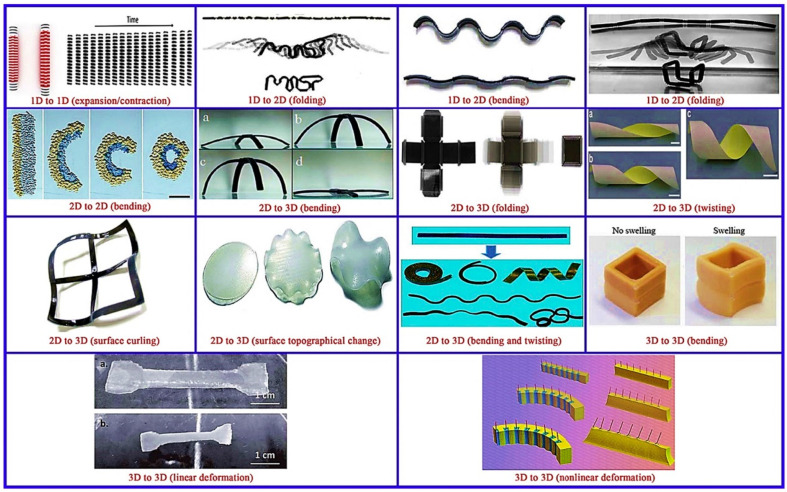
Various examples of different kinds of shape responses 1D to 1D (expansion and contraction) [25], 1D to 2D (folding and bending), 1D to 3D (folding) [55], 2D to 2D (bending) [56], 2D to 3D (bending) [57], 2D to 3D (folding) [55], 2D to 3D (twisting) [58], 2D to 3D (surface curling), 2D to 3D (surface topographical change) [55], 2D to 3D (bending and twisting) [59], 3D to 3D bending [60], 3D to 3D linear deformation [61], 3D to 3D nonlinear deformation [27]. Figures reproduced with permissions from [27,55,56,57,58,59,60,61,62].

To create consistently high-quality products, 3D printing requires high-quality materials that adhere to the strict requirements, much like any other manufacturing process. The procedures, requirements, and agreements about material controls are established between the suppliers, buyers, and end-users of the material in order to achieve this. Using a variety of materials, such as ceramics, metals, polymers, and their mixtures to create hybrid, composite, or functionally graded materials, 3D printing technology may create completely functioning components (FGMs). A comparison of the different 3D/4D printing technologies is presented in Table 1. As described earlier, a 4D printed object undergoes a shape transformation or change in the property when it is subjected to external stimuli such as water [24,25], light [26,27], heat [28,29], pH [30], electricity, magnetic fields, and so on [31,32,33,34]. The change in shape that may occur can be from 1/2/3D to 1/2/3D [54]. The examples of the shape transformations are shown in Figure 3.

### 2.2. Materials for 3D/4D Printing

Materials for additive manufacturing fall into three categories: polymers, ceramics, and metals. Polymers are the most regularly used and available materials, however, as technology advances, additional materials are being produced. Typically, materials are manufactured as wire feedstock or powders, however, this is diversifying over time. Due to the simplicity of producing and handling polymeric materials, 3D printing has traditionally concentrated on polymers for printing. However, the process has swiftly progressed to print not only diverse polymers but also metals and ceramics, making 3D printing a flexible production alternative. The primary uses, advantages, and difficulties of the primary materials for additive manufacturing are summarized in Table 2.

#### 2.2.1. Polymers

Due to their versatility and adaptability to various 3D printing techniques, polymers are regarded as the most widely used materials in the 3D printing industry. Thermoplastic filaments, reactive monomers, resin, and powder are the most common forms of polymers used in additive manufacturing. For many years, the 3D printing of polymers and composites has been investigated in a variety of industrial applications, including the aerospace, architectural, toy manufacturing, and medical industries.

In stereolithography 3D printing, photopolymer resins may polymerize when triggered by UV light. According to Wohlers Associates’ annual industry study, photopolymer-generated prototypes account for close to 50% of the 3D printing market in the industrial sectors [5,65]. The thermomechanical characteristics of photopolymers need still be enhanced, however, for instance, due to the gradient in UV exposure and intensity, the molecular structure and alignment of 3D-printed polymers depend on the layer thickness [66,67].

**Figure 4:**
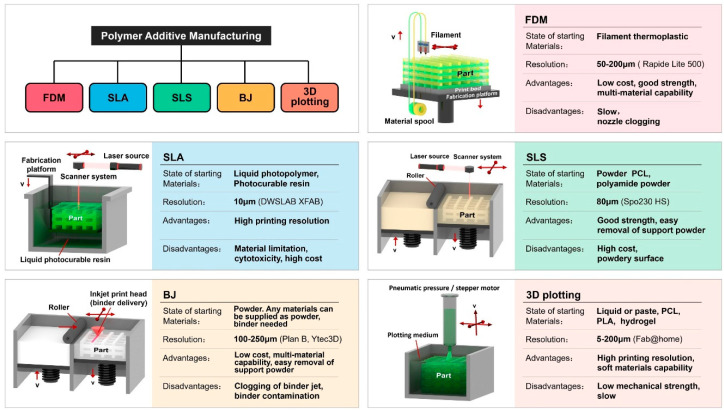
Various 3D printing technologies for polymer materials. Figure reproduced from [9].

There are several ways to produce polymer composites, including stereolithography, ink-jet 3D printing, selective laser sintering, and deposition molding [68]. Other approaches are either currently being researched and developed or are being used by scientists. Each method has pros and cons when it comes to the creation of polymer composite goods. The requirements for raw materials, processing speed and accuracy, cost, and product performance standards may all have an impact on the manufacturing process. Figure 4 displays the various available polymer 3D printing technologies.

Polydimethylsiloxane, also known as PDMS, is the most common kind of polymer used in silicone systems [69]. This is due to the material’s superior mechanical flexibility and stretchability, chemical inertness, biocompatibility, and high thermal stability when compared to other types of elastomers. In addition, PDMS maintains a significant degree of chemical stability even when subjected to high temperatures and pressures [70].

Thermosetting polymers go through chemical reactions throughout the formation process to create cross-linked structures, which harden after curing and do not melt when heated again. The thermosetting polymers can no longer soften when heated a second time because of this irreversible alteration. Phenolic plastics, amino plastics, epoxy plastics, unsaturated polyester plastics, organosilicon plastics, and polymethylmethacrylate (PMMA) are the principal types of thermosetting plastics [71,72].

The combination of nanoparticles with polymer materials may result in the production of high-performance functional composites. Lin et al. [73] demonstrated that the graphene oxide/photopolymer composite that was generated by SLA has both high levels of strength and ductility.

Nylon, polycarbonate, ABS (acrylonitrile butadiene styrene), and PLA (polylactide) including soft PLA, as well as recyclable alternatives, are all common polymers that may be used in 3D printing. Polymers like these are commonly utilized in material extrusion printers and are spread in wire feedstock. A number of approaches involve combining powdered polymers in varied amounts to achieve distinct aesthetic and structural effects. Waxes and epoxy-based resins are also often utilized.

Polymer additive manufacturing (PAM) may be used to produce delicate and complicated structures for the aerospace industry, structural models for the building industry, art replicas that are not real, and tissue and organs for use in medicine. However, the majority of AM polymer products are still employed as samples rather than practical components since they lack the strength and essential functionality of pure polymer products. The widespread industrial use of polymer AM is constrained by these drawbacks. Polymer matrix composites have been created by adding particles, fibers, or nanomaterial reinforcements to the polymer to address these drawbacks.

#### 2.2.2. Metallic Materials

Metals are employed in 3D printing complex electrical and circuitry components, as well as the structural and mechanical elements and integral functioning components. High-temperature processes can deposit them in liquid form, or they can be sintered or melted from powder. Steel, gold, silver, aluminum, cobalt-chrome alloy, titanium, and stainless steel are common metals used in 3D printing.

The process of 3D printing metals often begins with the melting of metallic feedstock, which can be either in the form of a powder or solid, with the use of an energy source such as a laser or an electron beam. The material that has been melted undergoes a transformation on a layer-by-layer basis to produce a solid component. Powder bed fusion (PBF) and direct energy deposition (DED) are the two methods that are most often used for the process of printing metals. PBF-based additive manufacturing procedures are capable of producing a wide variety of metallic materials, including stainless and tool steels, some aluminum alloys, titanium and its alloys, and nickel-based alloys, amongst others [74]. PBF technology can produce parts with excellent mechanical characteristics and intricate forms with a great precision.

AM has been optimized for titanium and its alloys, steel alloys, a few aluminum alloys, nickel alloys, and a few magnesium and cobalt-based alloys [74]. Particularly high-performance materials that are often utilized in a variety of sectors include titanium and its alloys [75,76]. They are distinguished by expensive machining and a lengthy lead time based on traditional production techniques. The ability of AM to produce very complex structures at reduced prices and with less waste may therefore result in major economic benefits. Materials such as austenitic stainless steels [77], maraging steels [78], precipitation-hardenable stainless steels [79], and tool steels [80] are often employed. In addition to the typical uses, these alloys may be utilized in situations requiring great strength and hardness, such as in the manufacturing of tools or molds.

For a variety of reasons, only a few Al alloys are presently utilized in AM. They are less expensive and easier to process than Ti alloys [81]. As a result, their usage in AM has drawn little commercial attention. This is mainly due to the reason that Al alone has a high reflectivity for the laser wavelengths often employed in AM [81], and certain high-performance Al alloys are rarely weldable because of the high volatility of some of its constituents, such as Zn [82].

**Figure 5:**
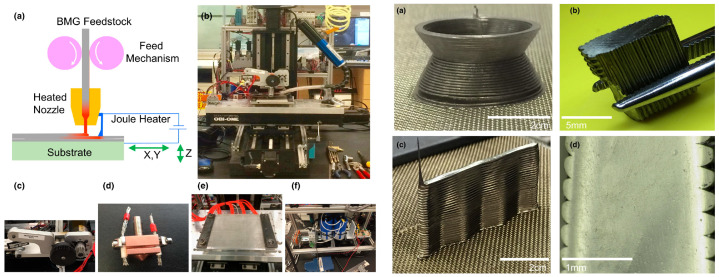
Example of metallic glass printing. The schematic and physical setup of fused filament fabrication (FFF) process for direct-write extrusion of bulk metallic glasses (**a**–**d, left**): (**a**) schematic, (**b**) physical setup, (**c**) feed mechanism, (**d**) extrusion nozzle, (**e**) heated substrate stage, and (**f**) capacitor bank that generate the applied volatage. The printed bulk metallic glass products (**a**–**d, right**): (**a**) continuous, (**b**) start-stop printing of BMG, (**c**) printed fully dense and pore-free parts, and (**d**) zoom in view of (**c**). Figures reproduced from [92].

Due to their randomly arranged atoms, metallic glasses (MGs) exhibit a wide range of intrinsic features, such as catalytic [83,84,85,86], soft magnetic [87,88], corrosion resistance [89], and excellent mechanical [90] capabilities. The majority of AM manufacturing processes include heating and cooling. The metallic glass starts to crystallize once it reaches the temperature for crystallization [91]. Both MGs and MG composites may be produced additively with a variety of characteristics for diverse purposes. Bulk metallic glasses (BMGs), in contrast to typical metals, exhibit a supercooled liquid zone and continuous softening upon heating, similar to thermoplastics. Gibson et al. [92] demonstrated that, by extending this analogy, BMGs may also be created via fused filament extrusion (FFF) in 3D printing. The supercooled liquid nature of the BMGs enables 3D printing under circumstances comparable to thermoplastics. In ambient environmental conditions, fully dense and amorphous BMG parts can be 3D printed which possess high strength as shown in Figure 5.

The production of complicated components built using expensive materials, such as titanium and its alloys, which are crucial for the aerospace and healthcare sectors, is made easier by additive manufacturing of metals. Metal additive manufacturing is a constantly expanding field, with new techniques, alloys, and applications being revealed increasingly on a regular basis. This leads to notable quality gains and faster production times.

#### 2.2.3. Ceramics

Typically, ceramics are utilized in powder-based additive manufacturing processes. Ceramic fibers are primarily made of silica and glass, porcelain, and silicon-carbide, and they can be coupled with polymer or metal substrate materials to boost their strength. A high mechanical strength and hardness, good thermal and chemical stability, and viable thermal, optical, electrical, and magnetic performance are some of the properties that make them such versatile materials for 3D printing. Ceramic components are typically formed into the desired shapes from a powder mixture with or without binders and other additives.

**Figure 6:**
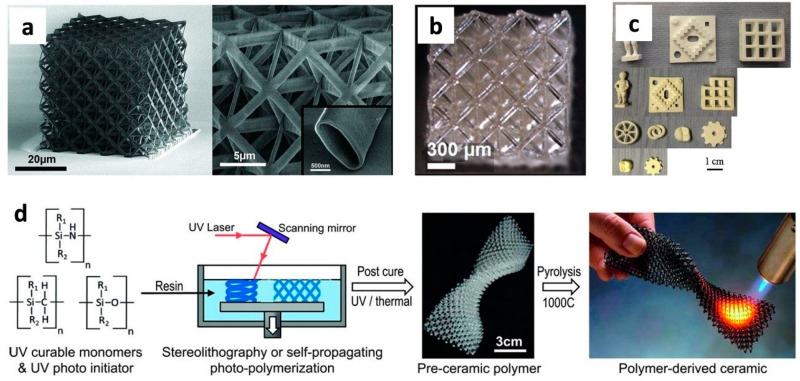
Examples of some common 3D printing procedures for ceramics. Small-scale ceramics may be 3D printed using coating-film-based ceramic printing feedstocks. (**a**,**b**): (**a**) an Al_2_O_3_ nanolattice with hollow tubes [104]. (**b**) Al_2_O_3_ hollow-tube micro-lattice [98]. (**c**,**d**) Large-scale ceramics printed in 3D: (**c**) selective laser sintering [95] and (**d**) stereolithography (SLA)/self-propagating photopolymer wave-guide technique for ceramics generated from polymers [93].

*Ceramics printing based on powder/slurry*. In comparison to polymers and metals, the AM of ceramics is difficult, owing to the exceptionally high melting temperatures of ceramic materials [93] and the difficult preparation of feedstocks [94]. Figure 6 depicts several common ceramic 3D printing procedures. Various AM techniques, such as SLS [95], selective laser burn-out [96], stereolithography (SLA) [97], projection micro-stereolithography (PµSL) [98], laminated object manufacturing (LOM) [99], DIW [100], IP [101], FDM [102], and digital light processing (DLP) [103], are commonly used to create ceramic structures from powders or slurries. Because of the significant heat gradients, many of these ceramic printing processes suffer from inescapable residual porosities and undesired fissures, resulting in poor mechanical behaviors of the manufactured ceramic structures.

*Coating film-based ceramics.* The invention of atomic layer deposition made it possible to cover TiN or Al_2_O_3_ on 3D printed polymer templates to create hollow ceramic nanolattices [98,104,105] or a ceramic composite microarchitecture [106]. After coating the ceramic sheets, the polymer template can be removed to reveal the delicate nano-/micro-structures. The microscale size of the created structures and the method’s poor manufacturing speeds, however, significantly restrict its use.

*Polymeric precursor-based ceramics.* Significant advancements in ceramic processing have been made possible by the AM of preceramic polymers. With just a slight and consistent shrinkage, printed polymers may be transformed in situ to ceramics, yielding intricate and exact ceramic constructions. Additionally, because pyrolysis temperatures are substantially lower than sintering temperatures, this operation uses a lot less energy than traditional powder or slurry sintering processes.

A revolutionary technique for creating highly shaped ceramic structures is the AM of ceramic precursors. For the last 50 years, polymer-derived ceramics (PDCs), created by the in situ thermolysis of preceramic polymers, have permitted enormous scientific advancements in the field of ceramics [94,107]. For a variety of structural and functional applications, PDCs have shown to be promising material choices [108,109].

One of the most promising preceramic precursors is silicon-containing polymers, which often include multinary ceramics like SiCNO [110,111] or ternary ceramics like SiOC [112,113] and SiCN [113,114]. PDC nanocomposites may be created by adding different kinds of nanofillers to preceramic polymers before machining, such as ceramics [112], metals [115], or polymers. These nanofillers can build jammed network structures in the preceramic polymer matrix to improve the mechanical integrity of the finished ceramics [116] and act as barriers to mass and heat transmission, reducing shrinkage during ceramization [107].

**Figure 7:**
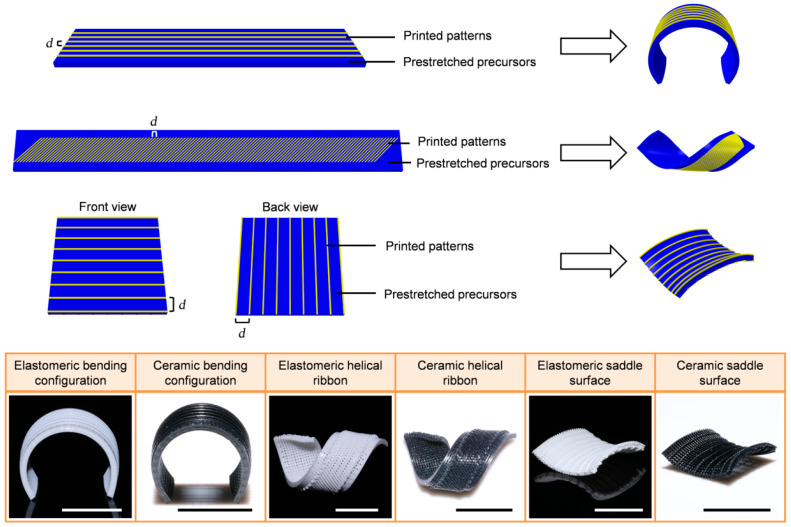
Four-dimensional printing of elastomer-derived ceramics (EDCs) and origami. Two typical ceramic 4D printing processes (scalebars: 1 cm). Figure reproduced with permission from [117].

Liu et al. [117] developed the first 4D printing ceramic, i.e., elastomeric poly(dimethylsiloxane) matrix nanocomposites (NCs) that are capable of being printed, deformed, and then changed into silicon oxycarbide matrix NCs, hence allowing the creation of intricate ceramic origami and 4D-printed ceramic structures, achievable as seen in Figure 7. By releasing the elastic energy trapped in the pre-strained ceramic precursors, which could be stretched to over a 200% strain, a shape-morphing process could be accomplished [117]. The above-mentioned research on elastomer-derived ceramics (EDCs) may provide new methods for creating soft/rigid hybrid structural materials, which may lead to innovation for the use of ceramic precursor/ceramic hybrid systems in a variety of fields, such as bio-implants and bio-inspired structures [118].

**Figure 8:**
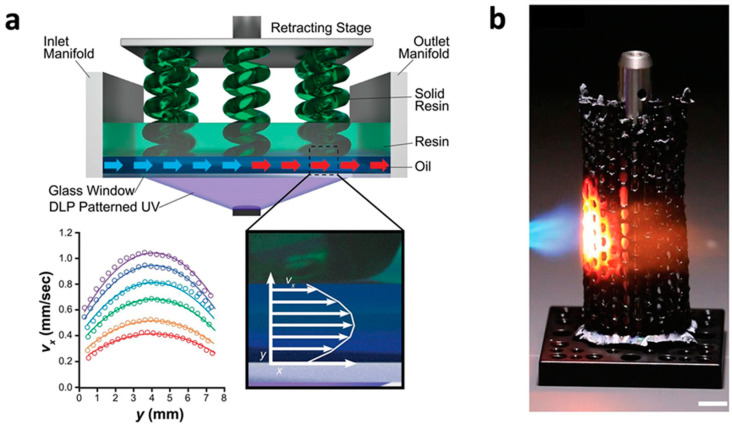
Examples of 3D printed polymer-based ceramics (PDCs). (**a**) High-area rapid printing (HARP) process diagram (**b**) HARP approach for the large-scale polymer-based SiC structures [41].

PDCs and EDCs will play an increasingly essential role in the additive manufacturing of ceramic structures in future research. Ceramic 3D printing is going to grow bigger and quicker as new printing material systems, printing methods, and post-processing techniques are developed as per a study presented in Figure 8 [41]. Ceramic 4D printing will make use of novel material systems, such as those with shape-memory properties, in order to become more adaptable, precise, and applicable.

#### 2.2.4. Other Novel Materials for 3D Printing

Theoretically, layer-by-layer printing may be done on any material. In order to create laminated objects, there are techniques to the 3D print adhesive and ordinary paper, chocolate, and sheets made of both polymer and adhesive. In principle, any material may be 3D printed; nevertheless, the high temperatures and pressures necessary by many additive manufacturing processes can fundamentally modify the microstructure of the material, resulting in changes in properties such as mechanical strength and durability after its creation.

Polymer carbon nanotube composites are produced when very trace quantities of carbon nanotubes are mixed with polymeric materials. This process results in the development of polymer carbon nanotube composites (PCNs). These nanotubes impart specific one-of-a-kind mechanical and electrical properties onto the PCN, which enables the material to take on a variety of various shape-changing actuation schemes [119]. This may be applied to produce a variety of PCNs, each of which is capable of exhibiting a distinct stimuli-responsive behavior, and as a result, this can be used for a broad number of applications. Polymers mixed with nanoparticles of iron oxide (Fe_3_O_4_) and carbon nanotubes have been utilized to generate a wide variety of different forms [120]. The incorporation of graphene nanocomposites into thermally activated polymers led to an improvement in the composite material’s tensile strength as well as an expansion of the range of deformation that could take place without the fibers breaking [121]. The use of graphene reduces the crosslinking density of the polymers, which results in the composite exhibiting an exceptional tensile strength.

## 3. Applications of 3D/4D Printing

### 3.1. Biomedical

The field of medicine has some of the most innovative and promising 3D printing uses. The process of “bioprinting” allows for the molecule-by-molecule construction of human tissues from stem cells. Three-dimensional printers have been used to make customized prostheses, drug delivery systems, scaffolds for tissue engineering, and implants. To offer biocompatibility or nanostructured programmable biodegradability, medical additive manufacturing employs polymers, metals, and ceramic materials as well as compounds with different doping agents.

According to a recent Wohlers’ research [65], the sector for additive manufacturing will increase from USD 6.1 billion in 2016 to USD 21 billion by 2020. The biomedical industry, which accounts for 11% of the entire AM market share today, will be one of the forces behind the development and expansion of AM.

**Figure 9:**
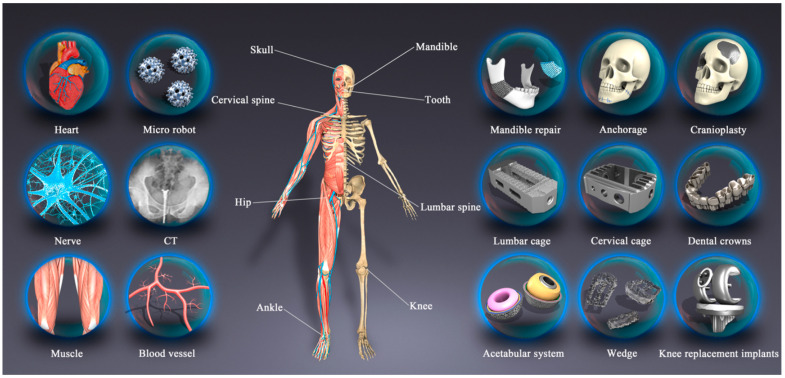
Application of AM in biomedical field. Figure adapted from reference [9].

A summary of the additive manufacturing applications in the biomedical field is presented in Figure 9. The field of dental implants and orthopedic prostheses has benefited from the AM application to manufacture hip, spine, ankles, skull, knee, and tooth components [122,123,124,125,126,127,128,129]. In tissue engineering and article organs, skeleton muscle, cardiac patches and hearts, and vascularization [44,130,131,132], in medical diagnosis and treatment for CT and MRI, and medical robotics [133,134,135] have used the applications of AM.

The use of AM for bio-implants has gained a growing interest. Compared with applications in other industries, medical implants have unique demands, including a high complexity, an excellent customization, and small production numbers, and hence, AM is ideally suited to this field [136]. Orthopedic prostheses and dental implants are made to be implanted into the body of a patient to heal shattered bones or teeth. Thus, they need a higher level of biocompatibility, adequate mechanical qualities, and excellent customization.

The chosen material must have comparable mechanical qualities and an acceptable biocompatibility. Metals, ceramics, and polymers are the three main materials used to treat bones and teeth. Traditional Ti-based materials are extensively employed in numerous research [137] and have a strong biocompatibilities and corrosion resistance [138], however, stress shielding causes aberrant bone development surrounding the implant [139]. In the manufacturing of implants, alloys with an excellent biocompatibility and biodegradation characteristics are gaining popularity. Ceramic materials are more appropriate for use in the biomedical sector since their mechanical properties and compositions are comparable to those of bone and teeth. Zirconia ceramic implants, such as teeth and crowns that are fabricated via 3D printing, have mechanical properties that are equivalent to those manufactured using the conventional techniques [140,141].

**Figure 10:**
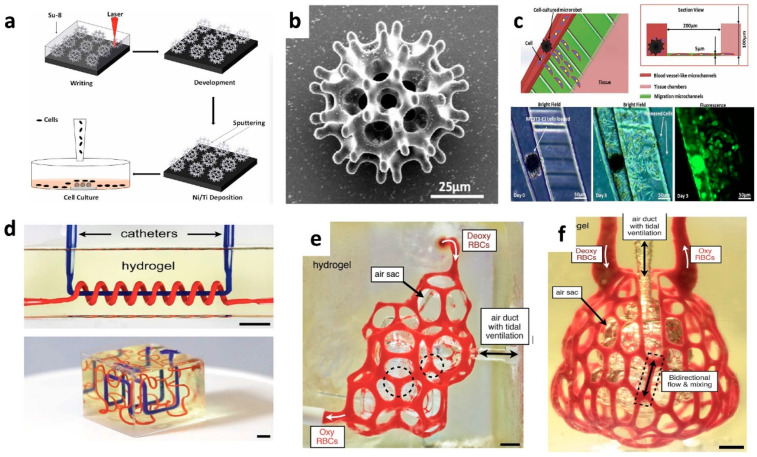
Applications 3D printing in the field of medicine. (**a**–**c**) Multi-vascular and intravascular networks printed by hydrogel networks for ventilation process and oxygenation [132] and (**d**–**f**) fabrication process and morphology of the magnetic micro-robot that can deliver cells [44].

Recently, multi-vascular and intravascular networks made of a biocompatible photopolymerizable hydrogel based on Poly(ethylene glycol) diacrylate (PEGDA) were created using an SLA technique [44]. The red blood cell flow and the oxygenation process were investigated (Figure 10a–c). The potential translational value of these biodegradable carriers in a model of chronic liver damage was also emphasized. Hearts and cardiac patches made of 3D printing have also been created [142]. The ability to print organs and tissues that are unique to a patient was proven by the printing of a heart with a natural structural design using a completely customized bio-ink.

In the realm of medicine, AM is a powerful instrument that may aid in the diagnosis of sickness as well as surgery. When it comes to planning and simulating surgery, specific 3D printed models of patients that are based on computed tomography (CT) or magnetic resonance imaging (MRI) scans may be helpful [143,144]. This is particularly true when it comes to the accomplishment of minimally invasive surgery. A burr-like porous microrobot was created by Li et al. [132] using laser lithography, and it was coated in Ni and Ti for magnetic actuation and biocompatibility (Figure 10d,e). This microrobot loaded with drugs can be transported and released to a specified location. The robot has a lot of promise for cell therapy and regenerative medicine (Figure 10f).

Jonathan and his colleagues [145] printed a bespoke head support, which had a number of benefits, including a reduced overall weight, a cheaper overall cost, and safer features. Because of the high resolution and the great efficiency of the AM technology, it is a powerful method that may be used for the creation of delicate and complex components. In the not-too-distant future, it will be feasible to create artificial tissues and organs that are tailor-made to individual specifications.

**Figure 11:**
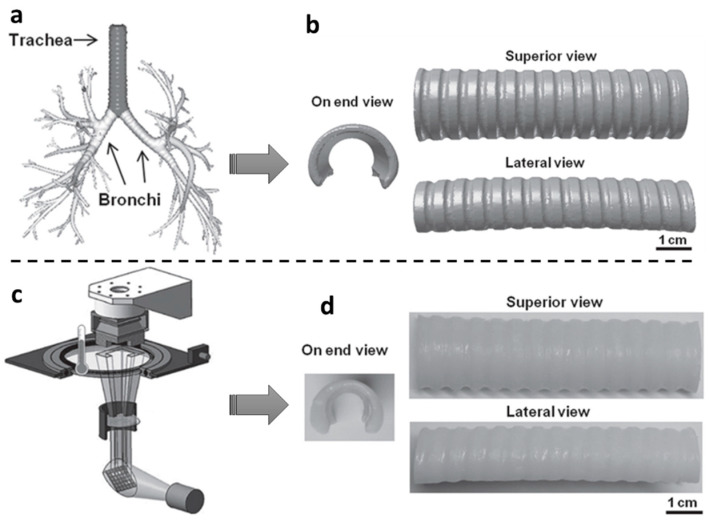
Developing process a shape memory airway stent, a tracheal stent used to alleviate breathing problems. (**a**) A digital model of a middle-aged male’s tracheobronchial tree obtained from MRI scan [146]. (**b**). A CAD model of the structure [146] (**c**) Using a specially designed heating bath that is heated over the melting point of the resin, the digital models are printed on a commercial SLA printer [147]. (**d**) The permanent shape of printed tracheal stent [146].

Four-dimensional printing can be used to develop adjustable stents that can change the shape or function in response to body temperature, addressing some of the issues associated with static stents. Zarek et al. [146] used 4D printing to build a thermo-responsive tracheal stent that could be twisted into a temporary shape, implanted into the body, and then deployed back into its original shape with the increase in the body temperature (Figure 11). It is safer and more advantageous to use than conventional stents since worries about stent migration and injuries sustained during insertion are eliminated.

### 3.2. Three-Dimensional Printing and COVID-19

The recent serious outbreak of COVID-19 all over the globe has had a significant effect on both the economy and society. At the beginning of 2020, COVID-19 significantly boosted the demand for essential healthcare supplies. Because of the need for a large number of items and the urgency of the situation, communities were compelled to turn elsewhere for temporary manufacturing solutions. This was made necessary by supply chain interruptions brought on by practical and political obstacles.

**Figure 12:**
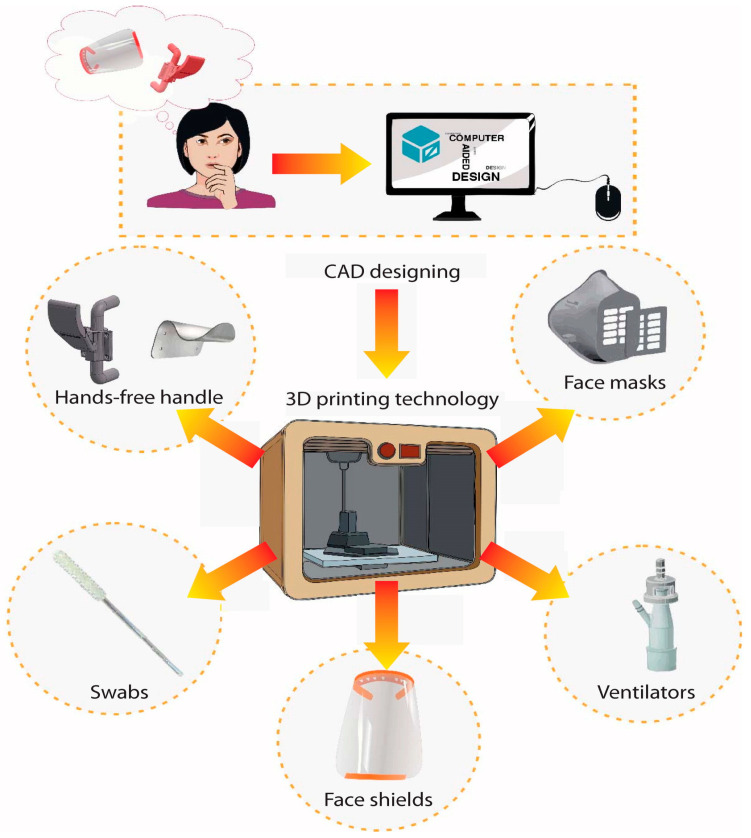
An example of the 3D printing process from conceptualization through computer-aided design modeling and manufacture of several pieces used in the COVID-19 pandemic defense. Figure reproduced from [149].

As a solution, global shortages of protective and medical equipment have greatly benefited from 3D printing’s accessibility and quick prototyping capabilities. People with access to 3D printers contributed to the efforts to combat the COVID-19 epidemic by conceptualizing and quickly developing preventative, diagnostic, and therapeutic devices (Figure 12) [148,149]. People from all over the world are coming together in online groups for this reason to discuss and trade designs and ideas.

Lu designed a whole new projection model for the COVID-19 pandemic [150] that was quite straightforward, and it has the ability to accurately anticipate both the outbreak and the trends in advance. The research and production of innovative face masks and other medical instruments, including certified respirators and virus screening gadgets, have a lot of promise thanks to AM technology.

**Figure 13:**
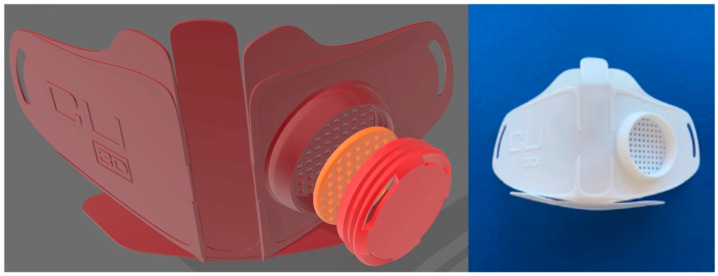
Model of the Copper3D NanoHack mask (**left**), showing a step in the creation of the mask. A 3D-printed replica of this mask (**right**). Figure reproduced from [149].

A NanoHack mask [151] was developed by the Copper3D company, which was intended to be manually assembled into its final 3D shape after being heated to a temperature of 55–60 °C (131–140 °F) by forced hot air (for example, a hairdryer) or by immersing it in hot water. This mask can be printed with polylactic acid (PLA) filament as a flat piece (Figure 13).

Masks and safety glasses may be printed from a variety of materials, including poly(ethylene terephthalate copolymerized with 1,4-cyclohexylenedimethylene terephthalate, or PETG), polyurethane (PU), and ABS. Utilizing 3D printing technology, masks and safety glasses with intricate designs and several functions may be produced. Meanwhile, a greater efficiency and lower costs may significantly minimize material waste. For those fighting an epidemic, safety glasses are essential since they have several drawbacks. The functionality and compliance issues with conventional protective glasses were resolved by researchers at Zhejiang University when they developed a platform for designing custom-made eyewear.

### 3.3. Aerospace

**Figure 14:**
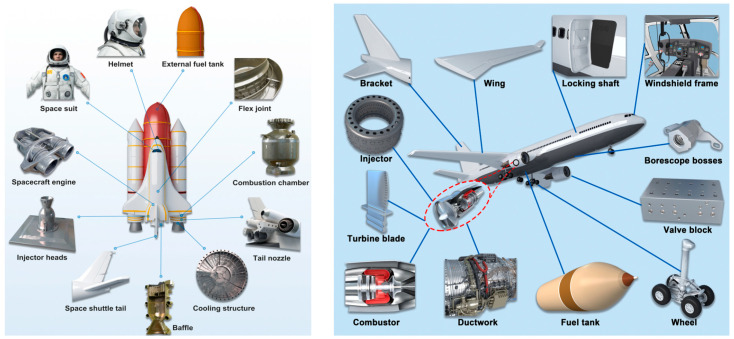
Applications of AM in the field of aerospace: astronautics (**left**), aeronautics (**right**). Figure reproduced from [9].

Many different industries are benefiting from the new opportunities that 3D printing presents, and the aerospace industry is one of them. Because of its advantages for design logistics, a high functionality, a high production efficiency, and lightweight products, additive manufacturing have extensively been used in the aerospace sector to make components. According to Wohlers’ analysis [65], the aerospace sector now accounts for 18.2% of the overall AM market and is regarded as one of the most promising industries in the foreseeable future. The current and future potential applications of 3D/4D printing in the aerospace field are demonstrated in Figure 14.

Aerospace applications that need metallic and non-metallic (such as metamaterials) [152,153] components may be created or repaired using AM. Some examples of these types of components are turbine blades, heat exchangers, and aero-engine components. The methods of additive manufacturing that do not involve the use of metal, such as stereolithography, multi-jet modeling [154], and FDM, are utilized for the rapid prototyping of parts, as well as the production of fixtures and interiors made of plastics, ceramics, and composite materials.

Astronautic parts such as engines, cooling structures, baseplates, baffles, and combustion chambers [155,156,157] are being manufactured by 3D printing technologies. While in aeronautics, several studies report the applications of 3D printing in the manufacturing of engines, brackets, spacer panels, valve blocks, tools, injectors, and chambers [158,159,160,161,162,163,164].

The manufacturing methods used by NASA now include a variety of different states and approaches of depositing material. NASA has an edge over other nations due to the fact that it started conducting research and tests several decades ago. It has focused much of its attention on metal and related materials.

**Figure 15:**
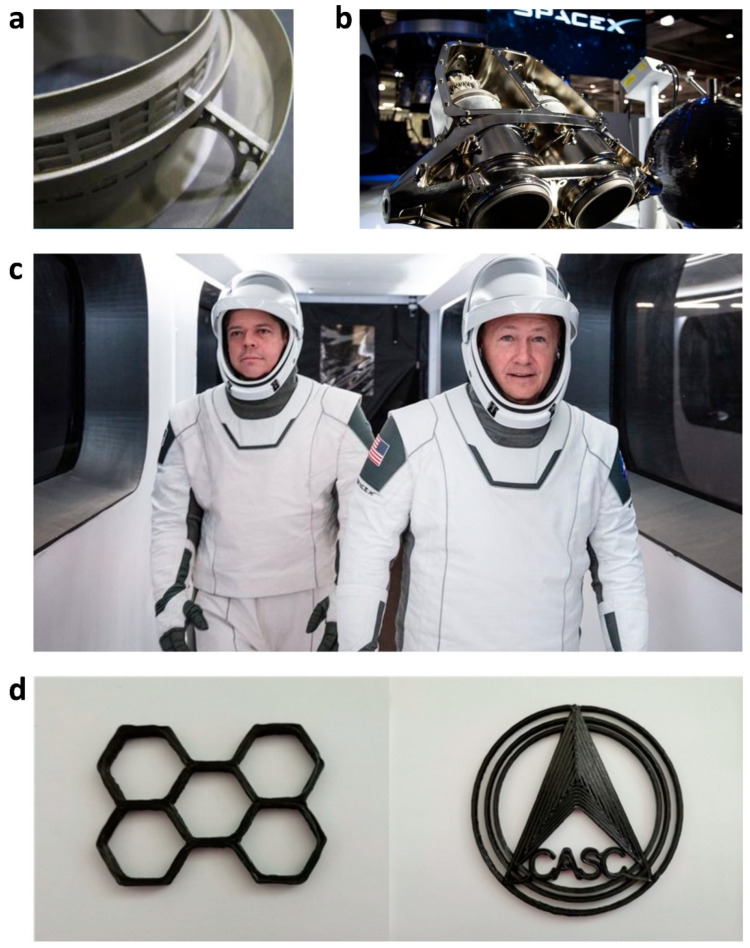
(**a**) The flex joint of the RS-25 [164], (**b**) SpaceX’s SuperDraco engine [165], (**c**) astronauts Bob Behnken and Doug Hurley wearing SpaceX spacesuits [165], and (**d**) two samples of continuous fiber-reinforced 3D printed components put into orbit by China: a honeycomb structure, and the China Aerospace Science and Technology Group Co., Ltd. Logo [166]. Figure reproduced from [9].

SpaceX’s manned Dragon spacecraft carried two astronauts, Bob Behnken and Doug Hurley, who donned 3D printed helmets for the space voyage (Figure 15). Along with the production of the Dragon V2 and Falcon 9 rockets, SpaceX also uses 3D printing for this purpose. The majority of the components of the two astronauts’ white helmets were created using 3D printing technology [164]. The “Chang Zheng 5B” carrier rocket from China has a 3D printer on board.

In addition to its industrial uses in the metallic and plastic engine and frame connecting parts, additive manufacturing has made a major contribution to the design of modern aircrafts, particularly with its morphing wings. The most crucial parts utilized to modify the flying states of aircraft are the wings, which are critical components that create lift forces for aircrafts. Morphing wings and adaptable structures are the most often chosen approaches to improve the existing aircraft with fixed wings, whose adaptive configurations are typically formed by mechanical rotations, in response to the growing need for the high mobility and fuel economy of modern aircraft. The common use of AM in conceptual research on morphing wings makes use of the great efficiency and low cost of 3D printing [167,168,169,170,171,172,173,174,175].

AM is utilized extensively in the aviation and aerospace sectors, which has sped up the design and manufacture of a number of different components. AM has made it feasible to manufacture designs that are less heavy, more robust, and more energy efficient. The production of engine components, aerostructures, airframe parts, aircraft parts and systems, interior parts, secondary structures, and avionics parts may all be accomplished using additive manufacturing on an airplane. AM is used in the production of both military and civilian engines, making this one of its most vital uses.

Because the aerospace industry often works on highly resistant and technically advanced components, and because additive manufacturing is the predominant method of production in this industry, metal seems to be an excellent option for the aerospace industry. Within this industry, the utmost importance is placed on both quality and safety. The 3D printing of metal is becoming more sophisticated, and the aeronautic industry has specific requirements and makes use of this expensive technology and materials.

Custom-made components and structures will further enhance the research on morphing wings and achieve the industrial manufacture of tailored morphing wings as AM technology develops and matures. This will be particularly true for multi-material AM and 4D printing. The morphing structures that have been accomplished will be of use not only in the commercial and military aviation sectors, but also in other areas of aerodynamic application, such as morphing automobiles and wind power generators. 

### 3.4. Flexible and Wearable Devices

**Figure 16:**
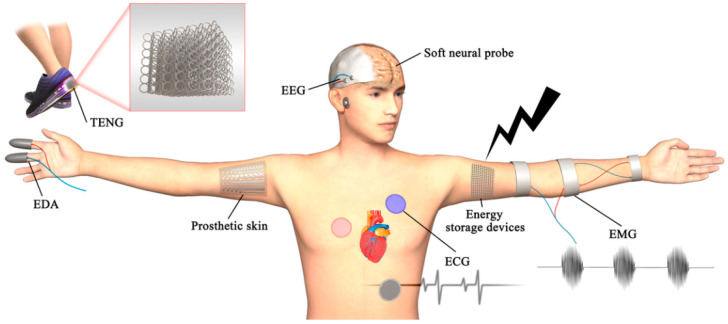
Applications of 3D printing in flexible and wearable devices. Figure reproduced from [9].

In recent decades, the interest in flexible electronics has increased. As shown in Figure 16, several published papers documented various flexible and wearable technologies, including sensors [176,177,178,179], nanogenerators [180,181,182], capacitors [183,184], Li-ion batteries [185,186,187], and flexible electrodes [188,189,190,191]. Flexible electronics may now be made using a variety of functional materials thanks to 3D printing’s high efficiency and resource-saving features. When it comes to structural materials, 3D printing not only guarantees the material’s performance but also enhances the resilience of printed materials. The development of several novel printed functional materials opens the door for flexible electronics to be used in wearable electronics. Additionally, several attempts have been undertaken to expand the applications for wearable electronics via the use of 3D-printed structures.

Wearable electrodes for electroencephalogram (EEG) signal measurements [189], prosthetic skin integrated with multimodal strain sensors [179], flexible electrodes for electrocardiogram (ECG) signal measurements [190], and 3D printed all-fiber Li-ion batteries for wearable electronics [185] are just a few of the recently published works that have focused on wearable electronics made using AM. 

**Figure 17:**
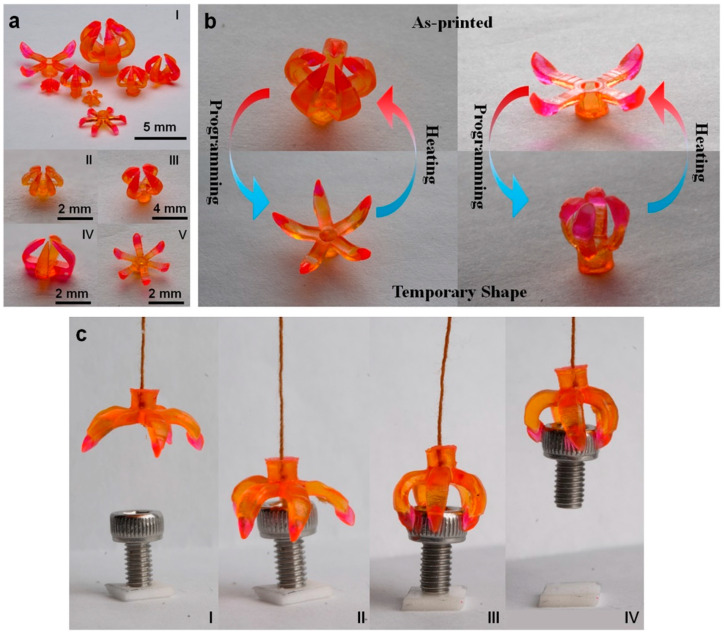
Different designs to construct multi-material grippers. (**a**) The illustration of the transformation of multi-material grippers from their printed shape to the temporary shape. (**b**) The transition of the grippers from their printed shape to their temporary shape upon heating. (**c**) The snapshots depict several stages of the process of gripping an object. Figure reproduced with permission from [197].

Due to its capacity to take on its original shape again, a shape memory-based triboelectric nanogenerator (STENG) is also suitable for soft robotic applications [192]. Using shape memory materials to create soft robotic grippers using 4D printing methods has been the focus of various studies [193,194,195,196]. These grippers are highly flexible and can handle a variety of objects responding to an external stimulus such as heat. Figure 17 is an illustration of a soft robotic system designed to perform certain gripping tasks.

### 3.5. Construction Industry

The use of additive manufacturing is currently expanding globally. Construction may now be done remotely and continuously using 3D printing technology for materials like concrete, glass, metal, and wood. It is necessary to have access to large-scale 3D printers in order to construct buildings and other infrastructure. These printers are capable of releasing rushes of concrete that harden in a short amount of time and can do so on a huge scale. Because traditional construction requires skilled workers to mix and pour down concrete, it is extremely laborious and financially crippling. However, because 3D printers can constantly form new materials for each piece of the project, construction workers and builders can use a single 3D printer to operate several projects at once, thereby minimizing the amount of time and money that is spent.

The use of AM in the construction industry is still in its infancy since it was only employed for residential construction for the first time in 2014 [198], but it has shown tremendous promise ever since that year. In recent years, there has been a rise in interest in the use of automated building construction techniques in conjunction with 3D printing technology. It has the ability to completely change the landscape of the construction business, and it might make building on the moon much simpler for astronauts [199]. It offers a considerable decrease in the amount of time and labor required for construction [200].

**Figure 18:**
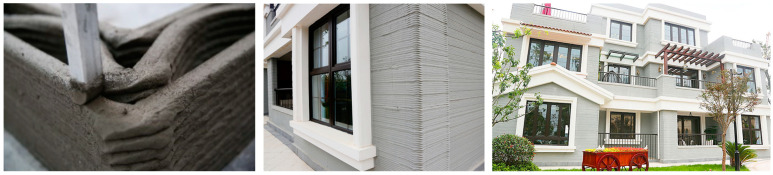
A 3D-printed three-story villa by Winsun in Wujiang, China. Figures reproduced with permission from [198].

In 2013, Winsun, a Shanghai-based construction company, created the first-ever batch of ten 3D-printed homes (Figure 18), after developing a 3D printer for the construction industry, making global headlines [198]. The printer adds layer by layer to create walls and other components in its facility in Suzhou (China) using a unique ink consisting of cement, sand, and fiber, along with a proprietary additive. The walls are then assembled on the spot. The first 3D-printed office building, which opened in Dubai in May 2016, was also the creation of Winsun.

Taking full advantage of the design freedom offered by the technology, the company has created a number of prototypes that customers can view outside of its main factories, such as a six-story apartment building, an affordable house, a wave-shaped house, or a traditional Chinese house in the old style. Winsun, however, has moved beyond prototyping and has so far sold more than 100 homes. Being the pioneer of 3D printing technology in construction, they faced a number of obstacles to scaling up their solution, including a lack of regulation and distrust from designers, project developers, and owners.

**Figure 19:**
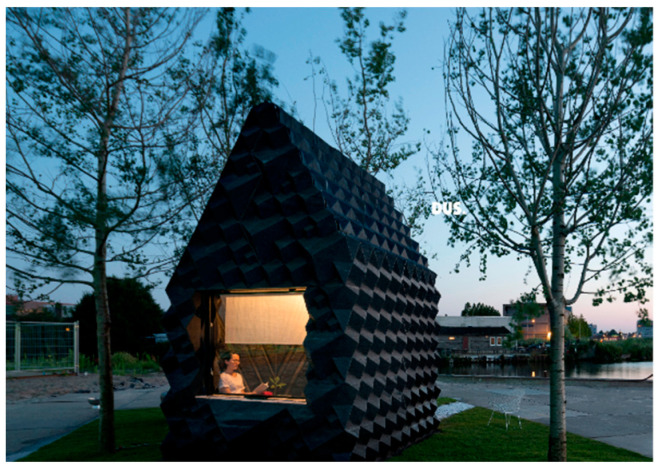
The first 3D-printed house by DusArchitects [201]. Figure reproduced with permissions.

A year later, in 2014, the city of Amsterdam saw the development of the first-ever residential building that was manufactured using 3D printing technology (Figure 19) [201]. The FDM technique was used. The project was pushed through by architects from Dus Architects, who intended to show the mobility of the printer with a low material loss and transportation expenses, in order to pave the way for it to be used in the construction business.

Three-dimensional printing has also emerged as an essential method for the replication and maintenance of cultural artifacts. Xu et al. [202] devised a method for duplicating a component of a historic building by integrating 3D scanning with cement mortar-based 3D printing. This method was used to reproduce the component. In comparison to more conventional approaches, building carried out with the aid of this technology resulted in significant savings in terms of both money and labor.

The need for qualified people with the capacity to combine robotic and civil work together is still a barrier for AM in the construction sector since the raw materials and manufacturing process used in 3D printing are different from traditional building techniques [203]. Although the use of additive manufacturing in the building sector is still in its infancy, it is clear that this technology has the potential to advance current construction methods. There will be additional possibilities and problems for the construction sector as a consequence of a further exploration and development of this technology.

### 3.6. Art and Jewelery

**Figure 20:**
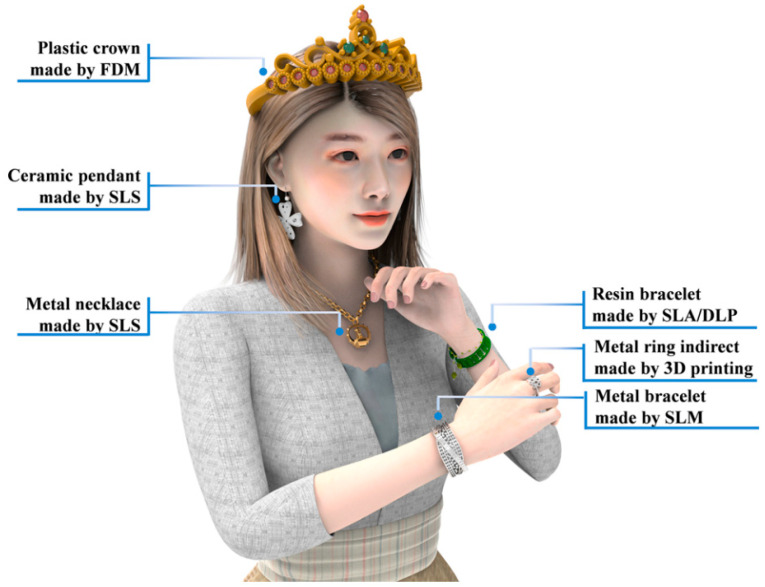
Examples of applications of AM in Jewelry and art decorations. Figure reproduced from [9].

Because of its fast manufacturing, customization, flexible design, and zero waste creation, 3D printing is quickly becoming a powerful technology that may be used in the production of art decorations and jewelry [204,205]. The manufacturing process for traditional jewelry and decorations must go through multiple processes, including manual carving, investment casting, polishing, and other fine surface processing. These processes take a significant amount of time and require a considerable effort, particularly the manual carving process [204,205]. By using 3D printing technology in the production of jewelry and other artistic embellishments, several laborious and time-consuming human procedures may be eliminated. As a direct consequence of this, jewelers are now selling an increasing quantity of jewelry and embellishments that have been manufactured using 3D printing technology. The most important uses of 3D printed jewelry and art embellishments are outlined in Figure 20, and the 3D printed versions may essentially serve as a replacement for conventional jewelry. Among the current applications of AM in jewelry and art decorations are ceramic pendants [206], the indirect fabrication of metal rings, resin bracelets and earrings [207], metal necklaces [208], and metal rings [209,210].

## 4. Challenges Ahead

The currently available methods for 3D/4D printing are limited in their abilities to create complex shapes. Overcoming this limitation would certainly increase the applications of 3D/4D printing in numerous industries. Although 3D printing has a highly promising future, there are still a number of challenges that must be resolved before the technology can advance. In comparison to traditional production methods, 3D printing is frequently slower and more expensive. Quality and sustainability are related considerations as well. The following problems need to be resolved in order for 3D printing to be even more practical in the future.

Faster Speed. Each object in a layer-by-layer manufacturing system requires a fixed amount of time to manufacture since the system has a predetermined completion time. However, following the initial creation of tools and molds, production may be automated and objects can be easily mass-produced with standard manufacturing technologies such as injection molding which will decreases the production time. 3D printing is preferable to the traditional methods created for mass manufacturing for short production runs. However, older techniques can readily outperform 3D printing in terms of scale.

Cost-effective. Due to the fact that only one or two machines are needed to print objects, 3D printing is less expensive than traditional production methods for small production runs. Moreover, there is also less waste produced with 3D printing than with traditional methods. Many times, additional tooling is not necessary with additive manufacturing. As a result, a 3D printer’s per-object cost is constant. A traditional mass production method, on the other hand, will use less energy over a shorter period of time to produce a greater number of items. The price of an item is typically not significantly more than the cost of the material needed to create it after the initial expense of establishing the requisite equipment. In time, a conventional process will be more cost-effective than using 3D printing equipment to create identical objects because of its energy efficiency and faster production rate. Depending on the objects being created, a 3D printer’s cost will eventually match that of utilizing a traditional technique. A traditional approach becomes more cost-effective per unit item than additive manufacturing at this “breakeven” threshold. This point was discovered to be inside the first 150 units of a production run in one Xometry investigation.

Better Quality. There is a structural flaw in the design since 3D printing layers are stacked one on top of the other. While there are several ways to design around this problem, for those producing very high-quality parts for industrial applications, quality might be a major worry. Another difficulty with layering for some 3D printing methods is that stripes created within an item may make it look less appealing. There are ways to finish 3D printed objects such that these layers are concealed, but they take more time and effort than the objects manufactured using other ways. The easiest way to address these challenges is to produce, at high resolutions, laser-based techniques. However, this causes items to take much longer to 3D print and, depending on the material, may still require an extra refinement to get the desired design and resolution. Furthermore, such laser-based systems are expensive, making them more appropriate for industrialized firms and huge corporations with substantial resources.

Higher sustainability. The speed of conventional production methods to manufacture identical structures may eventually surpass 3D printing for some objects. Therefore, the initial sustainability advantages of a 3D printer over the conventional methods are negated by the energy needed to power a printer and the requirement to refine materials into a usable shape. Thus, mass production of large quantities of objects using a traditional manufacturing method may still be more environmentally friendly in the long run until renewable energy and "greener" procedures become more common. Moreover, compared to the conventional methods for massive structures and buildings, 3D printers require vast amounts of energy to create houses and other large objects.

Another obstacle has been the inability of end-users to properly and meaningfully integrate additive manufacturing into their businesses due to the lack of expertise and knowledge. This has often confined the usage of 3D printing to the application of prototypes alone. Last but not least, the restricted selection of appropriate materials that are now accessible to end-users has traditionally prohibited the implementation of 3D printing in more exciting and demanding conditions.

## 5. Technology Advancement of 3D and 4D Printing

**Figure 21:**
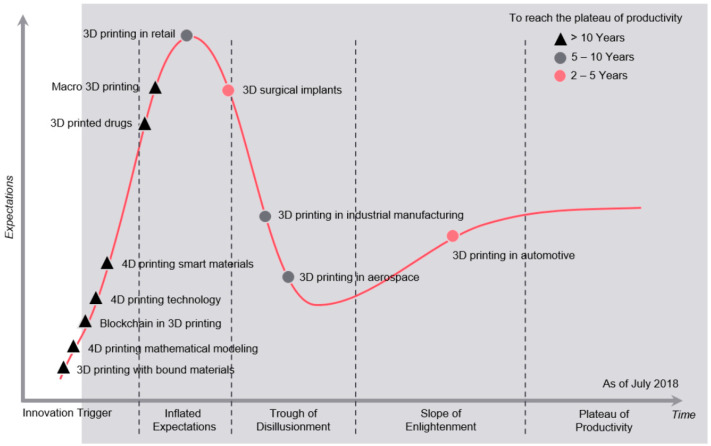
Gartner Hype Cycle of 3D and 4D printing technologies. Figure reproduced from FutureBridge Analysis [211].

The present stage of technological advancements in 4D printing is shown in Figure 21 below. The technology has undoubtedly generated a lot of hype while it is still in the development trigger stage, but it will take longer than 10 years to reach the productivity phase.

The hype cycle also shows that several advances in 3D printing are still in the innovation trigger and inflated expectations stages of their lifecycle. This implies that 3D printing has a bit of a way to go, and 4D printing, as the successor to 3D printing, may be slow to catch up. However, advancements in 4D printing do not always follow advances in 3D printing. Aside from a 3D printer’s capabilities (the ability to print multiple materials uniformly and on multiple axes), other research areas focusing on smart materials and mathematical modeling do not rely on 3D printing.

## Figures and Tables

**Table 1:** A comparison of additive manufacturing printing processes, materials used, applications, and pros and cons. Table adapted from [63,64].

Fabrication Process	Methods	Materials	Applications	Surface Finish	Merits	Limitations
Extrusion	FDM/FFF	Thermoplastics filaments, e.g., PLA, ABS, Nylon	Rapid prototyping Concept parts Advanced composite parts	Standard	Low cost Versatile Simplicity High speed	Weak mechanical properties Limited materials
	DIW	Plastics, Ceramics, Composites, Living cells	Packaging Scaffolds for bone regeneration	Standard	Flexible	Requires post processing
Powder-bed fusion	SLS	Fine powders of polymers, alloys, composites, and ceramics	Aerospace components Light-weight structures Electronics	Standard	Fine resolution High quality Best mechanical properties	Low resolution High cost High porosity
	SLM	Fine powders of metals, alloys, and ceramics	Aerospace components Light-weight structures Electronics	Good	Good mechanical properties Wide range of materials	Slow process
Photopolymerization	SLA	Photopolymers UV curable resins	Biomedical Prototyping	Excellent	High precision Smooth surface finish Low cost	Limited materials Weak mechanical properties Expensive
	DLP	Elastomers, Photopolymers UV curable resins	Biomedical Prototyping	Good	High resolution High printing speeds	Requires post processing

FDM, fused deposition modeling; FFF, fused filament fabrication; DIW, direct ink writing; SLA, stereolithography; SLS, selective laser sintering; SLM, selective laser melting; DLP, digital light processing.

**Table 2:** A comparison of the materials used in 3D/4D printing, their applications, benefits, and challenges [64].

Materials	Main Applications	Benefits	Challenges
Metals and alloys	Aerospace and Automotive Military Biomedical	Multifunctional optimization Mass-customization Reduced material waste Fewer assembly components Possibility to repair damaged or worn metal parts	Limited selection of alloys Dimensional inaccuracy and poor surface finish Post-processing may be required (machining, heat treatment or chemical etching)
Polymers and composites	Aerospace and Automotive Sports Medical Architecture Toys Biomedical	Fast prototyping Cost-effective Complex structures Mass-customisation	Weak mechanical properties Limited selection of polymers and reinforcements Anisotropic mechanical properties (especially in fibre-reinforced composites)
Ceramics	Biomedical Aerospace and Automotive Chemical industries	Controlling porosity of lattices Printing complex structures and scaffolds for human body organs Reduced fabrication time A better control on composition and microstructure	Limited selection of 3D-printable ceramics Dimensional inaccuracy and poor surface finish Post-processing (e.g., sintering) may be required
Concrete	Infrastructure and construction	Mass-customization No need for formwork Less labour required especially useful in harsh environment and for space construction	Layer-by-layer appearance Anisotropic mechanical properties Poor inter-layer adhesion Difficulties in upscaling to larger buildings Limited number of printing methods and tailored concrete mixture design
